# Targeting mitochondria to stimulate hematopoiesis

**DOI:** 10.18632/aging.102807

**Published:** 2020-01-28

**Authors:** Mukul Girotra, Olaia Naveiras, Nicola Vannini

**Affiliations:** 1 Laboratory of Immunosenescence and Stem Cell Metabolism, Department of Oncology UNIL CHUV, Ludwig Institute for Cancer Research Lausanne, University of Lausanne, Epalinges, Switzerland; 2 Swiss Institute for Experimental Cancer Research (ISREC), School of Life Sciences, Swiss Federal Institute of Technology Lausanne (EPFL), Lausanne, Switzerland; 3 Hematology Service, Department of Oncology, Centre Hospitalier Universitaire Vaudois (CHUV), Lausanne, Switzerland

**Keywords:** hematopoietic stem cells, mitochondria, NAD, mitophagy, asymmetric cell divison

Hematopoietic stem cells (HSCs) consist of a small cell-population in the bone-marrow (BM) that are responsible for lifelong production of all mature blood cells in an organism. A delicate balance of different HSC fates, namely, quiescence, self-renewal and differentiation is decisive in maintaining the HSC pool and blood cell homeostasis. Cellular metabolism has emerged as one of the fundamental regulators of HSC fate decision process. HSCs rely primarily on anaerobic glycolysis while downstream progenitors use mitochondrial metabolism to fulfil their energy requirements [1]. This distinct metabolic state protects the HSCs from cellular damage inflicted by reactive oxygen species produced by active mitochondria, thereby maintaining their long-term *in vivo* function [2] and possibly driving epigenetic modifications orchestrating HSCs fate determination. We and others, have previously shown that low mitochondrial activity is a hallmark of functional HSCs [1,3,4]. Additionally, we have demonstrated low mitochondrial activity as *conditio-sine-qua-non* required by HSCs undergoing self-renewing divisions in *ex-vivo* cultures [3].

In a recent study we have tested the mitochondrial modulator and NAD+ boosting agent, Nicotinamide Riboside (NR), in the context of regenerative hematopoiesis [5]. NR, together with nicotinic acid and nicotinamide, belongs to the vitamin B_3_ family and, differently from other analogs, NR, enters the NAD salvage pathway through its conversion into nicotine-mononucleotide (NMN) accomplished by the substrate-specific enzyme NR kinase (NRK). One week of NR dietary supplementation to wild type mice resulted in increased BM cellularity and expansion of hematopoietic progenitor cells, this reflected in a significant increase in terminally differentiated circulating blood and immune cells. Importantly, mitochondrial profiling of HSCs derived from mice supplemented with NR revealed significant reduction of mitochondrial membrane potential (an indirect readout on mitochondrial activity), indicating that NR has a direct effect on HSC metabolism when administered systemically. In order to examine if the effect of NR is cell autonomous, we exposed BM derived HSCs to NR in *ex vivo* cultures and found significant lowering of mitochondrial membrane potential (ΔΨm). Moreover, NR effectively reduced ΔΨm of fetal liver, cord-blood and adult aged BM-derived human HSCs, in a dose-dependent manner. Accordingly, in line with our previous published data [3], NR-induced reduction of human HSC ΔΨm reflected in an amelioration of their long-term human blood reconstitution capability.

To understand the molecular mechanisms driving the effect of NR, we performed transcriptome analysis (by RNA sequencing) on e*x vivo* cultured HSCs. We found upregulation of autophagy (and mitophagy) and of NAD salvage pathway genes upon NR treatment, and a concomitant downregulation of mitochondrial metabolism pathway genes (TCA cycle and Oxidative Phosphorylation). NR-induced mitophagy was confirmed by image analysis of *in vitro* cultured HSCs and by bone marrow analysis of mitophagy reporter mice (mito-QC). Importantly, the use of Mito-QC mice evidenced that NR exerts a direct effect on the most primitive hematopoietic compartment when the compound is administered systemically in the diet. We discovered that mitophagy induction was coupled with activation of mitochondrial unfolded protein response (UPRmt), that has been recently proposed as a conservation mechanism of HSCs pool during aging [6]. We hypothesize that NR-induced mitochondrial stress leads to the clearance of damaged mitochondria unable to coop with the metabolic stress. This complex mechanism initiates an instruction process where cells are primed toward asymmetric self-renewing cell division via differential distribution of active mitochondria in daughter cells.

Importantly, the resulting hematopoietic progenitor expansion occurring in mice supplemented with NR has critical clinical implications. Indeed, 20% of patients undergoing bone marrow transplantation die because of delay in blood and immune recovery. Given the dependency of blood and immune cell production on hematopoietic progenitor function, we asked if NR could boost hematopoietic recovery post BM transplantation and shorten the period of aplasia. In limiting cell dose transplantation setting, the control mice did not survive more than 4 weeks, while 80% of NR fed did. We found that this improved survival was due to a faster exit from neutropenia and thrombocytopenia in NR fed mice. Moreover, using Mito-QC mice, we demonstrated that under conditions of hematopoietic stress (transplantation), NR diet supplementation strongly induces mitophagy in hematopoietic stem and progenitor cells. These data indicate that mitophagy processes are both critical in homeostatic situation and in stress hematopoiesis context.

Given that previous studies have implicated the importance of mitophagy and autophagy in HSC function and aging [7,8] and our own finding showing that short *ex vivo* exposure to NR lowers ΔΨm in aged human HSCs, we believe that NAD boosting strategies could be used to improve functionality of the hematopoietic stem cell pool in elderly, where HSCs lose their capacity to produce a balanced immune system being strongly primed toward a myeloid fate (myeloid bias). Moreover, similar strategies could be employed in the context of myelodysplastic syndromes where hematopoietic defects are in certain cases associated with HSC mitochondrial dysfunctions.
